# Unique Mutation in SP110 Resulting in Hepatic Veno-Occlusive Disease with Immunodeficiency

**DOI:** 10.1155/2020/3460631

**Published:** 2020-01-10

**Authors:** Osama Hamdoun, Asia Al Mulla, Shamma Al Zaabi, Hiba Shendi, Sharifa Al Ghamdi, Jozef Hertecant, Amar Al-Shibli

**Affiliations:** ^1^Department of Academic Affairs, Tawam Hospital, Al-Ain, UAE; ^2^Department of Pediatrics, Tawam Hospital, Al-Ain, UAE

## Abstract

Familial hepatic veno-occlusive disease with immunodeficiency (VODI, OMIM: 235550) is a rare form of combined immune deficiency (CID) that presents in the first few months of life with failure to thrive, recurrent infections, opportunistic infections along with liver impairment. Herein, we are describing a Pakistani patient with a homozygous novel variant in the *SP110* gene, presenting with classical phenotypic manifestations of VODI. He presented at the age of 3 months with opportunistic infections and later developed liver failure. *Conclusion*. Hepatic veno-occlusive disease with immunodeficiency is a rare cause of immunodeficiency, and this is the first case report from the Middle East in a patient of Pakistani origin. It is important to have a high suspicion for this disease, in patients presenting early life with a picture of CID and deranged liver function, as the earlier the diagnosis and treatment, the better the prognosis.

## 1. Introduction

Familial veno-occlusive disease with immunodeficiency syndrome (VODI; Online Mendelian Inheritance in Man (OMIM) 235550) is an autosomal recessive immunodeficiency syndrome [1]. The key features of VODI include: (1) immunodeficiency (usually combined affecting the cellular and humoral function) and (2) liver involvement in the form of hepatomegaly with/without hepatic failure or histologically proved hepatic veno-occlusive disease (hVOD). Mutations in Sp110 gene leads to SP110 protein deficiency and the clinical manifestations of VODI [2].

Patients with VODI usually show manifestations early in life with repeated bacterial, viral, and fungal infections. These infections are usually serious and may be life-threatening. Herein, we are describing a case with homozygous novel variant in the *SP110* gene with classical phenotypic manifestations of VODI.

## 2. Case Presentation

This is a 3 month-old-male of Pakistani origin. He was delivered by normal vaginal delivery at term with an uncomplicated perinatal course. Parents are second degree relatives. He first presented initially to hospital at the age of 3 months with fever, oral thrush resistant to topical antifungal gels, and diarrhea.

Laboratory workup showed significant panhypogammaglobulinaemia with derangement of liver function test. Lymphocyte subset analysis showed moderate T-cell lymphopenia mainly affecting the CD4 population but was otherwise unremarkable (Table 1).

During his hospital stay, he suddenly developed acute respiratory failure, which required resuscitation and intubation. At that time, CXR showed bilateral ground glass appearance, which was suggestive of pneumocystis pneumonia (PCP) or cytomegalovirus (CMV) infection. CMV viral load was elevated of 39000; as a result, he was diagnosed with disseminated CMV infection and was treated with ganciclovir with excellent response; repeated CMV viral load after treatment is less than 5780 copies, which is undetectable. He had bronchoalveolar lavage which showed mixed upper respiratory flora. His lymphocyte subsets normalized with CD4 count of 1,800 cells Micro/L. In spite of normal lymphocyte subsets, the presence of disseminated CMV and panhypogammaglobulinaemia was highly suspicious of severe combined immunodeficiency (SCID). Unfortunately, lymphocyte proliferation studies were not available. The patient was therefore commenced on antimicrobial prophylaxis and immunoglobulin replacement therapy, and currently patient is not having any new infections.

Moreover, the patient developed recurrent ascitic fluid accumulation, hypoalbuminemia, and further derangement of liver enzymes. Investigations ruled out protein losing enteropathy and nephropathy.

Abdominal ultrasound showed slightly enlarged liver and moderate amount of ascites. Peritoneal fluid analysis was normal. Liver biopsy showed sinusoidal obstructive syndrome (veno-occlusive disease), portal and lobular eosinophils, and noncaseating granulomas. Currently, his liver disease is static, but it is expected to worsen overtime.

Table 1 shows the laboratory data of the patient at the time of the presentation.

Whole exome sequencing (WES) was suggestive of the hepatic veno-occlusive disease with immunodeficiency as it confirmed *SP110* (NM_080424.2) homozygous variant c.691 C > T p (Gln231*∗*). Null Mutation is expected to result in absent protein expression, and the disease is most likely from consanguineous parents Table 2.

## 3. Discussion

VODI was described originally in Australians of Lebanese origin by Mellis and Bale in 1976 [3]. A majority of children reported with VODI have been of Lebanese origin with prevalence of one in 2,500 [1].

**Figure 1:**
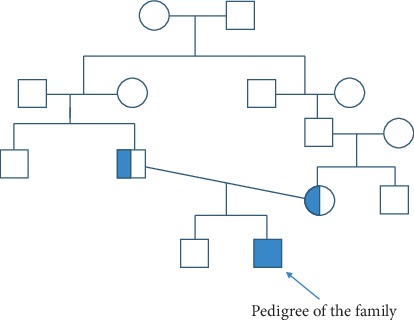
Pedigree of the family.

There were other reports afterwards from different regions of the world with novel mutations from families of Italian, Hispanic, and Arabic ethnic origins [2, 4] (Figure 1).

The age of presentation is around 4 months (usually before 12 months) with respiratory distress, fever, failure to thrive and diarrhea, as well as disseminated CMV infection, rotavirus-related gastroenteritis, and respiratory *Pneumocystis jiroveci*. Our patient presented at the age of 3 months with recurrent oral thrush and failure to thrive. At 4 months, he developed disseminated CMV infection with hepatitis and pneumonitis. In a group of 16 patients with VODI, clinical hepatosplenomegaly was detected in 12 patients at presentation [3, 4]. Ninety percent of the children with VODI present with either hepatomegaly (83% with preceding infection) or hepatic failure (53% with preceding infection) [2].

Neurological manifestations (occur in up to 30%) of cases are due to the veno-occlusive disease of the brain which may manifest as cerebral necrosis. Some of the patients described in the literature had cerebral leukodystrophy.

Thrombocytopenia and syndrome of inappropriate antidiuretic hormone secretion also have been described [5].

VODI is associated with 100% mortality in the first year of life if unrecognized and untreated with immunoglobulin replacement and *Pneumocystis jirovecii* prophylaxis, and a 90% mortality overall by the midteenage years if not treated with bone marrow transplant [5].

The immunodeficiency is characterized by severe hypogammaglobulinemia, clinical evidence of T-cell immunodeficiency with normal numbers of circulating T and B cells, absent lymph node germinal centres, and absent tissue plasma cells. Bacterial and opportunistic infections including *Pneumocystis jirovecii* infection, mucocutaneous candidiasis, and enterovirus or cytomegalovirus infections occur [1].

VODI is inherited in an autosomal recessive manner. Carrier testing for at-risk relatives and prenatal diagnosis for pregnancies at increased risk are possible if both pathogenic variants in a family are known [5, 6].

The *SP110* protein plays a crucial role in shaping the inflammatory milieu that supports host protection during infection by fine-tuning of NF-*κ*B activity which is required for normal T and B cell responses. A range of mutations in *SP110* causes decreased *SP110* protein levels and clinical disease. [7, 8] Some of the acquired immune deficiencies have been associated with veno-occlusive disease as well [9].

It is important to diagnose VODI in the first year of life as it is associated with 100% mortality if missed and untreated. There are reports of successful treatment of VODI with hematopoietic stem cell transplantation in few patients [10].

## 4. Conclusion

Hepatic veno-occlusive disease with immunodeficiency is a rare cause of immunodeficiency. To the best of our knowledge, this is the first report on a Pakistani patient with a novel homozygous mutation in *SP110* (NM_080424.2) homozygous variant c.691 C > T p. (Gln231*∗*) [11]. The patient was presented with classical presentation of liver impairment with immune deficiency.

## Figures and Tables

**Table 1:** Investigations at 3 months of age.

Investigation at age of 2-3 months	Result	Reference range
IgM	<0.25 g/l	0.15–0.70 g/l
IgG	<2.0 g/l	2.1–7.7 g/l
IgA	<0.06 g/l	0.05–0.40 g/l
CD3	1.404 cells/Micro/L	3.5–5 cells/Micro/L
CD4	0.744 cells/Micro/L	2.8–3.9 cell/Micro/L
CD8	0.637 cells/Micro/L	0.637 cells/Micro/L
CD19	0.581 cells/Micro/L	0.581 cells/Micro/L
NK cells	0.037 cells/Micro/L	0.1–1.3 cell/Micro/L
CD4/CD8	1.168 cells/Micro/L	0.9–3.6 cell/Micro/L
CMV quantitative	166,250	<490 cp/ml
WBC	17.5 × 10^9^	5–10 × 10^9^
Hgb	12.5	11–13 g/l
Platelet	67 × 10^9^	140–400 × 10^9^
Hepatitis B s Ab	Negative	
Hepatitis C Ab	Negative	
Hepatitis A IgM	Negative	
HIV	Negative	
BAL	Negative	
Karyotyping	46 + XY	

*Liver enzymes*		
Albumin	22 g/dl	
AST	217	22–58 IU/l
ALT	96	11–39 IU/l
Total bilirubin	22	5–22 micromol/l
PT	19.7	Seconds
PTT	46.9	Seconds

**Table 2:** Result of WES showing the defect of the *SP110.*

Gene	Variant coordinates	In silico parameters^*∗*^	Allele frequencies^*∗∗*^	Type and classification^*∗∗∗*^
*SP110*	Chr2(GRCh37):g.231076245G>A	PolyPhen: N/A	gnomAD :-	Stop gain
	NM_080424.2:c.691C>T	Align-GVGD: N/A	ESP :-	Likely pathogenic
	p.(Gln231^*∗*^)	SIFT: N/A	1000 G :-	(class 2)
		Mutation taster: N/A	CentoMD :-	
		Conservation: nt weak		

Variant description based on Alamut Batch 1.7 (latest database available). ^*∗*^Align-GVD: C0: least likely to interfere with function; C65: most likely to interfere with function; splice prediction tools: SSF, MaxEnt, and HSF. ^*∗∗*^Exome Aggregation Consortium (ExAC) database, Exome Sequencing Project (ESP), 1000Genome project (1000G), and CentoMD 4.0. ^*∗∗∗*^Based on ACMG recommendations.

## References

[1] Roscioli T., Cliffe S. T., Bloch D. B. (2006). Mutations in the gene encoding the PML nuclear body protein Sp110 are associated with immunodeficiency and hepatic veno-occlusive disease. *Nature Genetics*.

[2] Marquardsen F. A., Baldin F., Wunderer F. (2017). Detection of *Sp110* by flow cytometry and application to screening patients for veno-occlusive disease with immunodeficiency. *Journal of Clinical Immunology*.

[3] Mellis C., Bale P. M. (1976). Familial hepatic venoocclusive disease with probable immune deficiency. *The Journal of Pediatrics*.

[4] Cliffe S. T., Bloch D. B., Suryani S. (2012). Clinical, molecular, and cellular immunologic findings in patients with *SP110*-associated veno-occlusive disease with immunodeficiency syndrome. *Journal of Allergy and Clinical Immunology*.

[5] Tony Roscioli, Ziegler J. B., Buckley M., Wong M. (2018). Hepatic veno-occlusive disease with immunodeficiency. *GeneReviews*.

[6] Ivansson E. L., Megquier K., Kozyrev S. V. (2016). Variants within the *SP110* nuclear body protein modify risk of canine degenerative myelopathy. *Proceedings of the National Academy of Sciences of the United States of America*.

[7] Wang T., Ong P., Roscioli T., Cliffe S. T., Church J. A. (2012). Hepatic veno-occlusive disease with immunodeficiency (VODI): first reported case in the U.S. and identification of a unique mutation in Sp110. *Clinical Immunology*.

[8] Roscioli T., Ziegler J. B., Buckley M., Wong M. (2018). The responsible gene is *SP110* required for its transcriptional regulatory function and cellular translocation. *Journal of Biomedical Science*.

[9] Buckley J. A., Hutchins G. M. (1995). Association of hepatic veno-occlusive disease with the acquired immunodeficiency syndrome. *Modern Pathology*.

[10] Ganaiem H., Eisenstein E. M., Tenenbaum A. (2013). The role of hematopoietic stem cell transplantation in SP110 associated veno-occlusive disease with immunodeficiency syndrome. *Pediatric Allergy and Immunology*.

[11] Leu J.-S., Chang S.-Y., Mu C.-Y., Chen M.-L., Yan B.-S. (2018). Functional domains of *SP110* that modulate its transcriptional regulatory function and cellular translocation. *Journal of Biomedical Science*.

